# The NeuroImmunoEndocrine Circuit of Umami Peptides: A Systems Biology Approach

**DOI:** 10.3390/nu18081299

**Published:** 2026-04-20

**Authors:** Shiva Hemmati, Abdolali Mohagheghzadeh

**Affiliations:** 1 Department of Pharmaceutical Biotechnology, School of Pharmacy, Shiraz University of Medical Sciences, Shiraz 71345-1583, Iran; 2 Biotechnology Research Center, Shiraz University of Medical Sciences, Shiraz 71345-1583, Iran; 3 Department of Pharmaceutical Biology, Faculty of Pharmaceutical Sciences, UCSI University, Cheras, Kuala Lumpur 56000, Malaysia; 4 Department of Phytopharmaceuticals, School of Pharmacy, Shiraz University of Medical Sciences, Shiraz 71345-1583, Iran; mohaghegh@sums.ac.ir

**Keywords:** appetite, cognition, cytokine, GLP-1, gut–brain axis, memory, neuroendocrine, neuropeptide, synaptic plasticity

## Abstract

**Background/Objectives**: Umami peptides enhance flavor and contribute to appetite regulation (satiety) and metabolic health. By signaling to the orbitofrontal cortex, umami has been shown to improve cognitive function in Alzheimer’s disease dementia. This taste boosts the immune system and induces saliva secretion. However, the molecular mechanisms linking umami peptides to systemic physiology remain poorly understood. This study provides the first integrated analysis of neurological, immunological, and endocrinological pathways activated by umami peptides. **Methods**: Novel umami peptides were identified using machine-learning and deep-learning analyses from a library of marine-derived bioactive peptides. T1R1-T1R3 heterodimer is the dominant receptor for umami taste transmission in humans, expressed on taste cells, intestinal cells, and hypothalamic tanycytes. Molecular docking confirmed the binding of novel ligands to the T1R1-T1R3 receptor complex. New candidates and experimentally validated umami peptides, identified by sensomics approaches from tauco, chicken soup, pufferfish, and dry-cured ham, were analyzed using gene ontology. **Results**: The functional enrichment analysis revealed crosstalk among key signaling processes, including glutamatergic and opioidergic pathways. In addition to the role of µ1 opioid receptor (OPRM1), hub gene intersections highlight cholecystokinin (CCK), glucagon-like peptide 1 (GLP-1), and the anorexigenic pro-opiomelanocortin (POMC) neurons as potential regulators of the gut–brain axis in satiety signaling. Chemokine-encoding genes, melanin-concentrating hormone (MCH), oxytocin (OXT), and neurotensin (NTS) were other key target genes. **Conclusions**: The identified targets reveal the coordinated crosstalk between peripheral and central umami signaling that may contribute to the regulation of feeding behavior, satiety, cognition, memory, learning, and immune function. These network-based insights generate hypotheses and guide the design of nutritional and drug-like effectors for metabolic and cognitive health.

## 1. Introduction

High salt intake, particularly from hidden sources in processed foods, contributes to cardiovascular, renal, and gastric disorders [1,2]. Umami, the fifth basic taste, enhances saltiness and intensifies flavor [3]. Umami is characterized by a brothy, meaty, mouthful, or savory taste [4,5,6]. Free amino acids, peptides, nucleotides, Maillard reaction products, and organic acids might have umami tastes [7]. The first umami tastant, crystalline glutamate, was isolated from *Laminaria japonica* (now *Saccharina japonica*) by Ikeda [8]. Besides seaweed, sea urchin, crab, abalone, oyster, clam, prawn, dried skipjack, bonito broth, tomato, soy sauce, ripened cheese, cured ham, egg yolk, fermented soya bean, and shiitake mushroom are representatives of umami-containing compounds [6]. Therefore, monosodium glutamate (MSG) is approved as a GRAS food additive by the U.S. Food and Drug Administration (FDA) at normal dietary levels. However, the Federation of American Societies for Experimental Biology (FASEB) reported that consuming ≥ 3 g of MSG may cause mild symptoms, such as numbness, headache, tingling, flushing, drowsiness, and palpitations [9,10]. Natural umami peptides at specific concentrations can reproduce savory intensity at safe concentrations, providing alternatives to MSG [11].

The umami receptor is a G protein-coupled receptor (GPCR) composed of the taste receptor type I (T1R1) and type III (T1R3) subunits. The T1R1-T1R3 heterodimer is expressed on type 2 taste cells, intestinal I cells, and hypothalamic tanycytes [12,13]. Receptor expression across multiple organs translates umami signals into hormonal and neural responses that regulate feeding behavior and energy balance. For instance, activation of the T1R1-T1R3 receptor influences the secretion of gut satiety hormones such as cholecystokinin (CCK) [14]. Satiety is the transient loss of interest in further nutrient intake, and the signals are transmitted through the gut–brain vagal axis to the nucleus of solitary taste (NST) [15]. Umami has been linked to improved cognition in dementia, reflected by clear speech, eye opening, cheerful face, and satiety [16,17]. This taste enhances the immune system and stimulates saliva secretion, thereby maintaining oral mucosal integrity in patients with dry mouth and facilitating chewing and swallowing [18]. These physiological processes highlight umami’s integration within the neuro-immuno-endocrine system.

**Figure 1:**
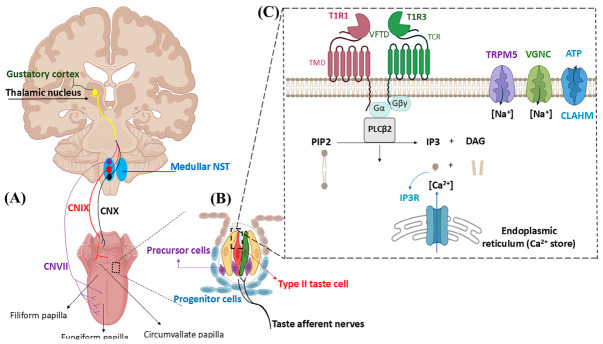
A schematic representation of the gustatory signaling pathway, taste bud cells, and umami taste detection by T1R1-T1R3 receptor [23,30]. (**A**) Sensory signals from different taste regions of the tongue are transmitted to the medullary NST. (**B**) Keratinocyte basal progenitor cells give rise to taste cell precursor cells, which differentiate into type I, II, and III taste cells. (**C**) Binding of an umami peptide to the VFTD of T1R1-T1R3 dissociates gustducin G protein and activates phospholipase C β2 (PLCβ2). The resulting inositol triphosphate (IP3) binds to ER receptors, causing Ca^2+^ release into the cytosol. The transient receptor potential action channel subfamily M (TRPM5) is then activated by Ca^2+^. Na^+^ ion influx through TRPM5 induces voltage-gated sodium channel (VGNC), depolarizes the membrane, opens calcium homeostasis modulator (CALHM), and triggers ATP secretion, which signals gustatory afferent nerves.

The primary umami signaling initiates from taste buds distributed on the papilla, soft palate, and pharynx (Figure 1A). Basal progenitor cells differentiate into type I, type II, and type III taste cells [19]. Type II taste cells express the T1R1-T1R3 chemosensory receptor complex (Figure 1B) [20]. Each subunit of the receptor contains an extracellular lobe called the Venus flytrap domain (VFTD) linked to a transmembrane domain (TMD) via a cysteine-rich domain (CRD) (Figure 1C) [21]. The binding of umami substances induces VFTD conformational changes, causes taste cell depolarization, and triggers ATP release, activating neural signaling via presynaptic type III cells [22]. Afferent nerve fibers innervate the base of taste buds to form synapses. The generated electrical impulses are transmitted to the brain via cranial nerves VII (CN7), IX (CN9), and X (CN10) (Figure 1A) [23]. Umami taste signaling in humans is ipsilateral via CN7 and CN9. Gustatory afferent nerve fibers converge at the medullary NST. Signals are then projected to the thalamus and primary gustatory cortex (PGC). Subsequent signaling to the orbitofrontal cortex (OFC), cingulate gyrus, lateral hypothalamus, amygdala, or basal ganglia is involved in taste-directed behavior and cognition [12,23].

Despite their key role in the gut–brain communication axis, the molecular mechanisms linking umami peptides to systemic physiological outcomes remain underexplored. Previous studies have focused on taste perception or receptor binding, but few have examined how umami peptides modulate gene networks that connect neural, endocrine, and immune responses. This study addresses the gap using a systems biology framework to elucidate interconnected signaling pathways. We screened a recently identified pool of marine bioactive peptides [24] to discover novel umami peptides using artificial intelligence. The interaction of novel umami peptides with the T1R1-T1R3 receptor complex was analyzed by molecular docking. A network pharmacology and gene ontology (GO) pipeline was then applied to identify systemic targets and functional enrichment profiles.

In addition, experimentally validated umami peptides (verified by sensory panels or electronic tongues) were used as positive controls [25,26,27,28,29]. This approach clarifies the connections among umami peptides, gastric secretion, feeding behavior, satiety, cognition, and immunity. In other words, the identified pathway crosstalk in this study links gustatory perception to systemic gut–brain–immune physiology.

## 2. Materials and Methods

### 2.1. Selection of Umami Peptides

To identify novel umami peptides, we screened a bioactive peptide library from 82 marine sources in our previous study [24]. Of 3505 peptides, 279 safe candidates (non-toxic and non-hemolytic) were analyzed to introduce putative umami peptides. Peptides predicted to be allergic or prone to in vitro aggregation were excluded. Three independent predictors were used to identify umami peptides. The selection of the three umami peptide predictors (UMpred-FRL, Umami-MRNN, and VirtuousMultiTaste) was primarily based on their availability as accessible tools in recent literature and their high-performance metrics, including sensitivity, specificity, and accuracy. UMpred-FRL (https://pmlabstack.pythonanywhere.com/UMPred-FRL, accessed on 28 January 2026) is an SVM-based meta-predictor combining seven different features to achieve a balanced accuracy and sensitivity of 0.860 and 0.786, respectively. It generates a probabilistic umami score (0–1 scale) from peptide sequences in FASTA format [31]. Peptides with a probability score higher than 0.5 are reported as umami using UMpred-FRL. Umami-MRNN (https://umami-mrnn.herokuapp.com/, accessed on 28 January 2026) is a deep-learning (DL) neural network predictor with an accuracy, sensitivity, and specificity of 90.5%, 95.2%, and 88.5%, respectively [32]. The program accepts FASTA input sequence files. Umami-MRNN provides a predicted concentration threshold (in mmol/L), representing the minimal peptide concentration required to elicit a detectable umami taste, allowing estimation of peptide potency. VirtuousMultiTaste (https://virtuous.isi.gr/#/virtuous-multitaste, accessed on 28 January 2026) was the most accurate predictor with 96% accuracy. This program combines heuristic optimization and nonlinear machine learning (ML) classification to distinguish umami peptides from other taste classes. It accepts SMILES, FASTA, or PDB inputs and provides results as text and spider charts [33]. VirtuousMultiTaste predicts multiple taste modalities simultaneously, reporting the probability of umami, bitter, and sweet tastes as percentages. The program uses heuristic optimization to efficiently explore feature and parameter combinations, improving prediction accuracy without requiring exhaustive computation. The primary marine-derived peptide database contained 10-, 15-, and 20-mer peptides, but most umami peptides are shorter than 15 residues in nature. Thus, only 10-mer peptides identified as umami by all three tools were nominated and selected for GO analyses.

### 2.2. Ligand–Receptor Interaction by Molecular Docking

Molecular docking was conducted to reconfirm the interaction between the newly identified umami peptides and the umami taste receptor. Predicted umami ligands identified by ML and DL methods were modeled using the PEP-FOLD3 program [34]. Since the X-ray crystallography of the human umami receptor is not yet available, T1R1 (Entry: Q7RTX1) and T1R3 (Entry: Q7RTX0) sequences were retrieved from the UniProt database. The T1R1-T1R3 heterodimer was modeled using the SWISS-MODEL program with the metabotropic glutamate receptor (mGLuR) (PDB ID: 1EWK) as a template [35,36]. Global model quality estimation (GMQE), QMEANDisCo global score, and sequence identity were three factors considered for selecting the most qualified model. Additionally, the PDB file of the selected model was submitted to the RamPlot program to generate a Ramachandran plot and assess the model’s geometric quality [37]. The fact that mGlu receptors serve as useful templates for G-protein coupled receptor (GPCR) modeling stems from their structural conservation of the transmembrane helical bundle despite moderate sequence identity [38]. The ligand-binding site was determined using the P2Rank program [39]. This step reduced bias associated with blind docking and focused subsequent simulations on biologically plausible interaction regions. The predicted binding sites were reconfirmed by comparison with previously reported umami receptor binding sites [40,41,42,43,44]. Molecular docking was performed using ClusPro 2.0 under default parameters in the standard balanced scoring mode, which incorporates van der Waals, electrostatic, and desolvation energy contributions [45]. The docked complexes were clustered based on RMSD, and the most representative structures from the top-ranked clusters (based on cluster size and weighted energy score) were selected for further analysis. ClusPro 2.0 was selected for peptide docking because it is optimized for protein–protein and peptide-protein interactions via Fast Fourier Transform (FFT)-based global sampling and cluster-based pose selection, which is well suited to flexible peptide ligands. This approach allows identification of energetically favorable and conformationally consistent binding modes in receptor extracellular domains [46]. To further validate the robustness of the docking results, the best ligand–receptor complexes obtained from ClusPro were re-docked using Molecular Operating Environment (MOE). Docking simulations in MOE were conducted using the standard docking workflow under default parameters unless otherwise specified [47]. Receptor structures (.pdb format) were first prepared using the QuickPrep module, which automatically corrects structural issues, assigns protonation states, adds hydrogen atoms, and performs restrained energy minimization using the Amber force field to relieve steric clashes while preserving backbone geometry. Putative binding sites were identified using the Site Finder module, which detects alpha spheres corresponding to potential ligand-binding pockets. The top-ranked site, based on pocket size and geometric suitability, was selected as the docking region. For each ligand, multiple poses were generated, and the top-ranked conformations were retained based on docking score (kcal/mol) and geometric plausibility. Ligand–receptor interactions were subsequently visualized and analyzed in MOE using the interaction fingerprint and 2D/3D contact mapping tools to identify hydrogen bonds, hydrophobic interactions, salt bridges, π–π interactions, and electrostatic contacts.

### 2.3. Identification of the Target Genes

**Figure 2:**
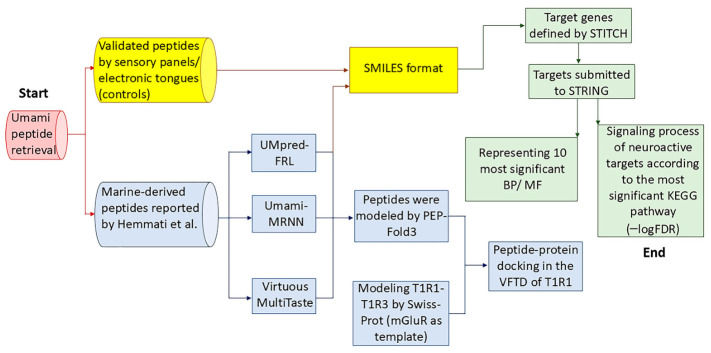
A flowchart summarizing the methodology applied in this study to find umami peptides and functional enrichment analyses. Bioactive marine-derived peptides were screened using machine-learning and deep-learning algorithms, validated through molecular docking, and analyzed via network pharmacology [24]. SMILES-converted peptides were queried in the STITCH database to identify target genes, followed by STRING-based protein–protein interaction, gene ontology, and KEGG enrichment analysis. A functional interpretation of hub genes, using GeneCards^®^ and literature mining, revealed the key signaling circuits mediating umami-induced neuro-immuno-endocrine regulation. BP: biological process, MF: molecular function.

The identification of molecular targets followed by a network pharmacology workflow is a standard approach to elucidate the mechanism of bioactive compounds [48]. This approach integrates chemical-protein association prediction, protein–protein interaction, and enrichment analysis. The resulting gene networks are analyzed for enriched biological processes, molecular functions, and KEGG pathways. This strategy identifies hub genes and core signaling circuits underlying peptide-driven systemic effects [49]. Accordingly, a network pharmacology approach was applied to define how umami peptides influence the expression and co-expression patterns of target genes, as described previously [50,51]. In addition to the novel identified peptides, six experimentally validated umami peptides were used as positive controls. Positive control peptides were selected based on predefined criteria to ensure structural and functional comparability with the newly identified marine-derived peptides. First, controls were chosen from protein-rich animal sources (fish, ham, chicken) to align with the marine origin of the novel peptides. Given the well-documented abundance of potent umami peptides in fermented foods, one peptide derived from fermented soybean was also included. Second, control’s peptide length was mainly in the range of 8–11 amino acid residues to match the predominant length of the newly identified 10-mer peptides and to minimize structural bias in downstream enrichment analyses. Third, only experimentally validated umami peptides with reported sensory thresholds determined by trained panels or electronic tongue analysis were included. Therefore, an umami peptide derived from fermented soybean (known as tauco, miso, doenjang, or dajiang in Indonesia, Japan, Korea, and China, respectively) has been designated control 1 in this study [27]. Other umami peptides originated from chicken soup (control 2) [25], Xuanwei ham (controls 3 and 4) [28], Jinhua ham (control 5) [29], and pufferfish (*Takifugu rubripes*) muscle extract (controls 6) [26]. The BIOPEP-UWM was used to convert peptide sequence to the simplified molecular input line entry system (SMILES) (https://biochemia.uwm.edu.pl/biopep/finding_smiles.php, accessed on 28 January 2026) [52]. The use of SMILES-based input in STITCH enables the retrieval of interaction data for the query molecule through a combination of direct matching and similarity-based mechanisms. Therefore, SMILES sequences were submitted to the STITCH database (version 5.0) (chemical structures tab) to identify target genes and interacting chemicals specifying Homo sapiens as the target organism [53]. In STITCH, similarity-based inference is not solely based on simple structural resemblance; rather, it integrates experimental interaction data, text-mined associations, and predicted protein-chemical interactions to infer potential protein targets. For bioactive peptides, particularly short food-derived peptides, experimentally validated target data are often limited. Therefore, similarity-based inference provides a practical and commonly used systems-level strategy to identify biologically plausible interacting proteins. The matched chemical structure with the maximum Tanimoto score was processed further. Default “Settings” parameters were retained except for specific updates. The active interaction sources included experiments, text mining, databases, co-expression, neighborhood, gene fusion, co-occurrence, and predictions. An interaction score threshold of 0.15 was selected to maintain sensitivity for food-derived peptides with limited experimental annotations, while restricting network expansion to 50 interactors per shell to preserve biological interpretability and prevent network overinflation. Target molecules were exported as TSV files (“Tables/Exports” tab). Protein identifiers were imported into the STRING database version 12.0 (“Multiple proteins” tab) to construct interaction networks and perform functional enrichment analysis [54]. GO analysis is a bioinformatics method that annotates genes and defines the biological processes, molecular functions, and related biochemical pathways [55]. All enriched GO terms were downloaded from the “Analysis” tab as a TSV file. GO terms were ranked by the lowest false discovery rate (FDR) values. FDR is a statistical value representing the rate of errors identified by the ratio of false positives to the sum of false positives and true positives. FDR is used for a high number of variables, retrieved for comparison via high-throughput technologies [56]. An FDR ≤ 0.001 was considered significant in this study. The ten most significant biological processes or molecular function terms (based on −logFDR) were displayed as cluster bar plots. GeneCards^®^ (the human gene database) was used to retrieve detailed functional annotations of the enriched genes. A targeted literature survey complemented database information to validate the biological relevance of the computationally predicted targets and pathways [57]. The workflow summary of the methods is illustrated in Figure 2.

## 3. Results and Discussion

Foods are a rich source of bioactive peptides with diverse benefits for human health [58]. The umami taste has evolved to detect proteins [23]. Besides oral receptors, GI distributed umami receptors regulate gastric emptying, protein digestion, and plasma amino acid levels [59]. Satiety perception upon ingestion is not only through taste sensation but also from GI processing transmitted to the CNS via gastric, celiac, and hepatic visceral vagal afferents [60]. However, the mechanisms underlying the integration of peripheral and cephalic umami sensations remain unclear. This study addresses the gaps by identifying target genes and performing GO analysis. Screening a library of safe marine bioactive peptides [24] with three predictive models identified four 10-mer umami peptides (Table 1).

### 3.1. Ligand Characterization and Ligand–Receptor Interaction by Docking

**Figure 3:**
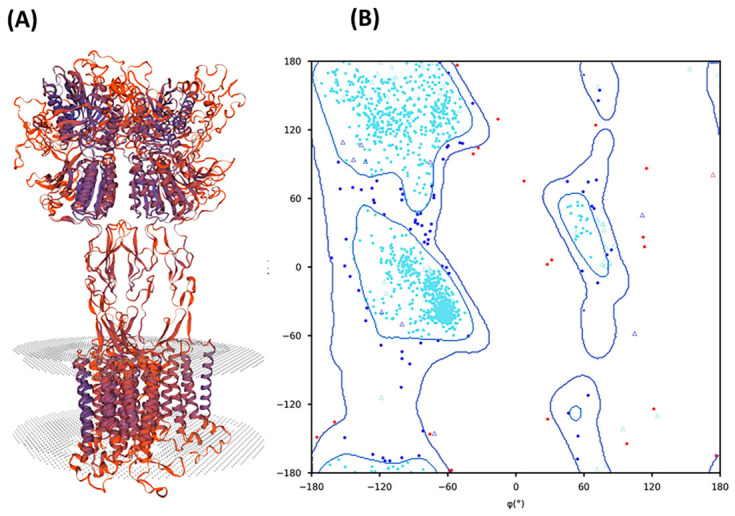
The 3D model of human T1R1-T1R3 umami taste receptor and the standard 2D Ramachandran plot. (**A**) The receptor is modeled using the SWISS-MODEL program using mGluR (PDB ID: 1EWK) as the template. (**B**) Ramachandran Plot analysis shows that 93.13%, 5.55%, and 1.32% of the residues are positioned in the most-favored (cyan), allowed (blue), and disallowed (red) regions of the plot, respectively.

Amino acid composition and the peptide’s physiochemical characteristics can determine taste sensation. Umami peptides are typically non-hydrophobic. Asp (D) and Glu (E) acidic residues are sometimes called indispensable residues for umami taste [61]. All four novel umami peptides contain D or E residues and are cationic with a low hydrophobicity between 10 and 20%. Cationic residues, such as Arg, also appear in clam (edible bivalve mollusk) and peanut hydrolysate umami peptides [62,63]. Although Cys does not directly confer umami taste, it contributes to the meaty perception and is found in Parma and Jinhua ham umami peptides [29]. Ser and Thr, as sweet residues, can increase the umami intensity. The synergy of umami and sweet residues reduces the bitterness of Phe, Trp, and Ileu [64]. Overall, amino acid composition is more determinative for short-chain peptides. The spatial structure is more critical for longer peptides [65]. Therefore, the ML- and DL-identified peptides were modeled and docked with the umami receptor for interaction analysis. The T1R1-T1R3 receptor was modeled using the Swiss-Prot program (Figure 3A). Analysis of the model’s quality showed that the QMEANDisCo and GMQE values for the selected model were 0.57 ± 0.05 and 0.31, respectively. QMEANDisCo is a structural quality metric that evaluates the actual 3D model by comparing inter-residue distance relationships against those derived from experimentally solved protein structures. The observed value suggests that the overall fold and internal distance constraints of the model are moderately similar to high-resolution structures, but also indicate some deviations likely due to structural differences between the template metabotropic glutamate receptor (mGluR) and the target. The mGluR templates are only about 35% identical in sequence to the T1R1-T1R3 target. mGluR structures have been widely used as starting points for homology modeling of the GPCRs, such as T1R1-T1R3, particularly when no experimentally resolved structure of the target receptor is available [38]. Ramachandran plot statistics reconfirm the model’s optimal geometry, with more than 93% of residues in the most favored regions of the plot (Figure 3B).

The P2RANK program was used to identify critical residues in the umami receptor binding pocket for rigid docking. Identified residues in the VFTD region of the human T1R1 subunit (hT1R1-VFTD) included S148, T149, A170, S172, S173, I189, D218, D219, Y220, E301, A302, and I326. Because T1R1 is the primary binding site for umami peptides, and T1R3 plays a modulatory role, stabilizing the heterodimer [66]. Asp218, Asp219, and Glu301 are introduced as the main binding sites in the literature [40,41,42,43,44].

**Figure 4:**
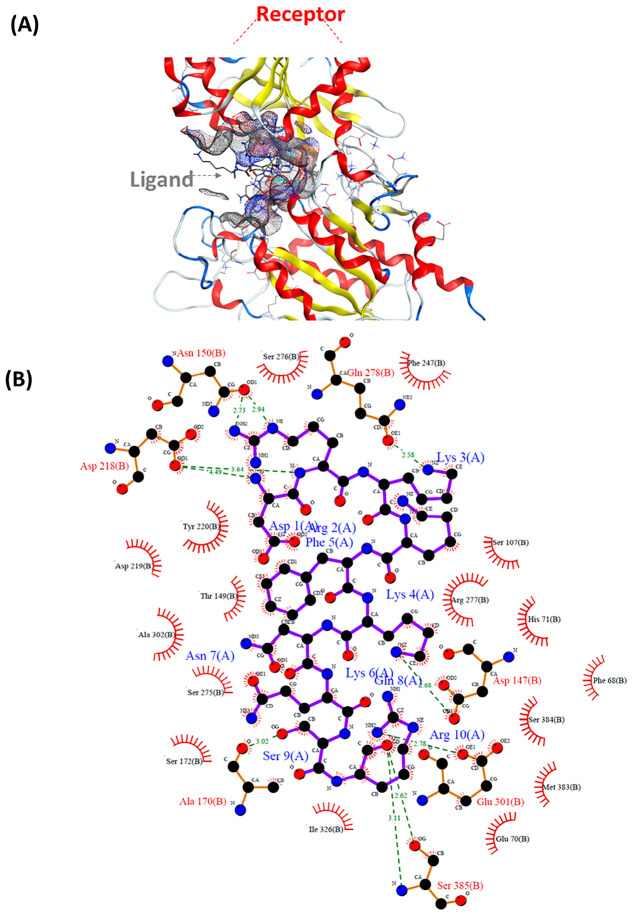
Molecular docking of the umami peptide, L10-40, as ligand and umami receptor. (**A**) The docked complex of L10-40 as the ligand with the VFTD of T1R1. (**B**) Ligand–receptor interactions in the binding pocket.

L10-40 showed 31 interactions with hT1R1-VFTD and the strongest binding affinity among candidates (Table 1) (Figure 4A,B). A total of 22 ligand–receptor interactions were designated as hydrogen bonds with distances ranging from 2.5 to 3.5 Å, which represents the stability of the complex (Table 2). ASP218 formed three ionic interactions (due to its acidic and polar nature) and six hydrogen bonds. Glu301 contributed four ionic and four hydrogen bond interactions. Gln278 showed the lowest ΔGbind (−12.2 Kcal/mol) due to interaction with Lys3 in the ligand. Gln278 interaction was also reported for umami peptides derived from the hydrolysates of *Hypsizygus marmoreus* (an edible mushroom) [65]. Asp1 (D1) anchors L10-40 to Asp218 on T1R1 through H-bond and ionic interactions (Table 2).

L10-352 formed 20 interactions with T1R1-hVFTD (16 H-bonds and four ionic) (Figure S1). Asp218 and Asp219 form the binding site core, and Arg151 is a key partner. While Asp218 showed the strongest affinity (−13.4 Kcal/mol), Argg151 H bonding has also been reported for fermented grain wine umami peptides [64]. The side chain amino group of Lys9 (K9) in L10-352 anchors the peptide via multiple ionic and H bonds with Asp218 and Asp219 (Figure S1). L10-683 and L10-1653 formed 28 and 25 interactions with the receptor’s binding site, respectively. Arg277 formed hydrogen bonds with L10-683 and L10-1653 (Figure S1), similar to beef and mushroom umami peptides [65,67]. Arg2 (R2) in L10-683 forms multiple strong H-bonds and ionic clusters with Asp218. The C-terminal residue in the ligand (Arg10) forms powerful H-bonds and ionic interactions with Asp147. Glu7 (the acidic residue) in L10-683 and Arg277 (the basic residue) on the receptor form a reverse salt bridge. L10-1653 binding is driven by electrostatic interactions of R3, R8, and R9 with receptor acidic residues (Figure S1). Arg9 (R9) in the ligand forms an H bond (−10.7 kcal/mol) and an ionic contact (−6.7 kcal/mol) at a perfect distance (2.72 Å), locking the peptide to Glu301. Arg3 (R3), as a major N-terminal anchor, displays electrostatic attractions to the receptor’s acidic pocket Asp218. Gln7 (Q7) also contributes key polar stabilization in the peptide’s mid-region. The side chain of R8 interacts electrostatically with Asp147 on the receptor.

Notably, molecular docking was used only as a supportive assessment to discuss interactions at the atom level, rather than as the primary validation framework of the study. The core identification of candidate umami peptides was based on three independent predictive models using ML- and DL-based programs, which integrate several features for peptide identification. These features are multi-dimensional, whereas docking evaluates only a narrow structural aspect. However, for improved structural accuracy, advanced protein structure prediction methods such as AlphaFold [68,69] and D-I-TASSER [70] could be applied to compare their resulting models with the SWISS-MODEL structure. A comparative evaluation of confidence scores and structural validation metrics would provide a more robust assessment of model reliability. Finally, the observed interactions in the docking process of our study are consistent with previously reported experimental and docking studies. This cross-validation with the literature strengthens the biological plausibility of the structural results.

### 3.2. Target Identification and Functional Enrichment Analysis

Proposing pathways from target-gene networks illustrates a systems biology perspective. This approach emphasizes organized molecular interactions rather than isolated gene expression fluctuations [71]. The target genes of each umami peptide were identified, and the most significant biological processes, molecular functions, and KEGG pathways were defined (Tables S1 and S2). Subsequent subsections describe the most significant target genes for novel and validated umami peptides across gustatory, endocrine, neural, and immune systems (Table 3 and Table 4).

#### 3.2.1. L10-40 Umami Peptide Targets

**Figure 5:**
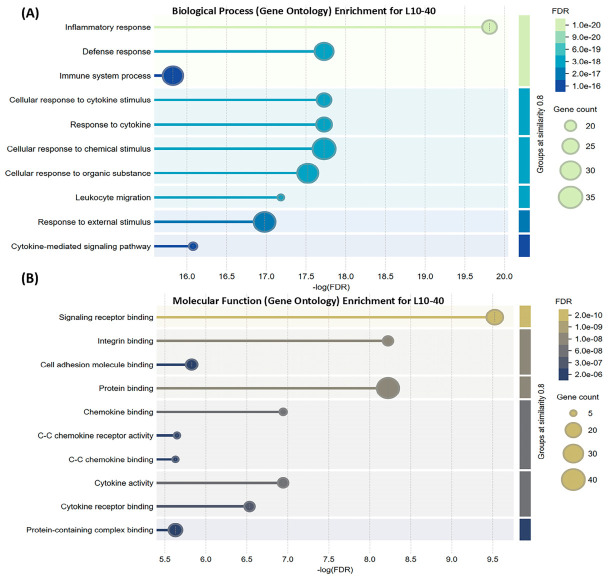
Functional enrichment visualization predicted for L10-40 umami peptide using the STRING V 12.0 database analysis mode. (**A**) Biological processes. (**B**) Molecular functions.

GO term analysis shows that L10-40 is associated with biological processes that are mainly related to “cellular response to immune system processes”, “defense responses”, and “cell adhesion”. The most significant (−logFDR) biological processes are represented in Figure 5A. Target gene functions are organized by immune signaling proximity. Target genes are chemokine-encoding genes and their corresponding receptors that initiate signaling. Some hubs are involved in adhesion and migration machinery (*ITGAL, ITGAM, ITGAX, ITGB2, VCAM1, CD44*). Other targets include membrane or cytoplasmic signaling mediators (*CD40, SRC*) and transcriptional regulators that integrate signals (*NFKB1, RELA, FOS, JUN, IRF1, TP53*). Finally, downstream targets and mediators of immune and inflammatory responses (*IL1B, IL6, IL10, TNF*) are observed.

The immune system components not only interact with each other but also with other organs and body processes [72]. Taste buds are influenced by external stimuli. Therefore, they are protected by the components of the innate immune system [30]. L10-40 is predicted to influence chemokines, such as *CCL2* and *CCL5*, and their cognate receptors (*CCR2* and *CCR5*). These ligand–receptor interactions initiate downstream signaling associated with inflammatory and defense responses. Chemokine-CCR signaling recruits and anchors immune cells. CXCL8 (IL-8) recruits leukocytes to the oral cavity (Table 3). Leukocytes adhere to the epithelium via cell adhesion molecules (CAMs) [73]. CAMs mediate cell–cell and cell-extracellular matrix interaction and organize taste buds [74]. L10-40 is associated with gene networks involving various *CAM*s, including integrin (*ITG*) subunits (Table 3, Figure 5B). Interaction of CAMs mediates chemotaxis, immune cell migration, monocyte adhesion, and phagocytosis [75,76,77]. CAMs also contribute to the conversion of progenitors to specialized taste cells and to their renewal [78]. *CD44*, another CAM, is expressed in taste bud progenitors and influences taste cell regeneration, maintenance, and signaling [79]. The interaction between taste cells and neural cells for signal transduction and processing is also mediated by neural CAMs (NCAM). NCAMs influence synaptic stability, umami taste strength, and perception [80]. FOS is a marker of neural activation in NST, cortex, and amygdala, where it modulates taste-driven learning [81]. FOS also regulates immune balance and macrophage phenotypes [82,83].

The expression of *TNF-α* and *IL-10*, as proinflammatory and anti-inflammatory cytokines, reflects the activity of *NF-κB* and *IRF1*. The balance of cytokines is vital for taste bud homeostasis and protection of peripheral taste structures [84]. TNF-α induces *CAM* expression, which facilitates leukocyte attachment to endothelium and chemokine secretion by phagocytic cells [85]. The predicted target profile of L10-40 overlaps with immunomodulatory pathways reported for miso, a fermented soybean paste, with a strong umami taste. Miso enhances communication between immune cells and intestinal epithelial cells (IEC) and upregulates *IL-10*, *IFN-γ*, and *IL-1β* [86]. Anticancer activity against gastric, breast, and colon cancer has also been reported for miso. Similar immunostimulatory effects have been reported for *Sparassis crispa* fermented with lactic-acid bacteria, resulting in enhanced umami intensity. This preparation increases IL-10, TNF-α, and IL-1β levels, induces immune cell accumulation in the jejunum and spleen, increases *CCR2*-expressing cells, and triggers phagocytic activity against *E. coli* [87]. FGF2 limits excessive inflammation by reducing proinflammatory cytokines such as TNF-α, IL-1β, and IL-6 [88,89,90]. Together, these findings suggest that L10-40 may be involved in networks related to chemokine-receptor interactions, adhesion molecule dynamics, and transcriptional pathways. These cascades are associated with cytokine-related signaling and cell-adhesion pathways implicated in immune protection and taste bud integrity. The dual umami-active and antimicrobial potential of L10-40 warrants further study.

#### 3.2.2. L10-352 Umami Peptide Targets

L10-352 is associated with biological processes related to the neuropeptide signaling pathway, feeding behavior, regulation of secretion, and digestion (Table S1). Table 3 lists targets of L10-352. The target genes are organized by functional and signaling proximity, including central satiety hubs (*POMC*), gut chemosensing genes (*CCK, GRP, HRH2, NPY4R*), reward and motivation circuits (*HCRT, OPRM1, NPFF*), adrenergic modulation (*ADRA1A* and *ADRA2B*), and inflammatory signaling.

**Figure 6:**
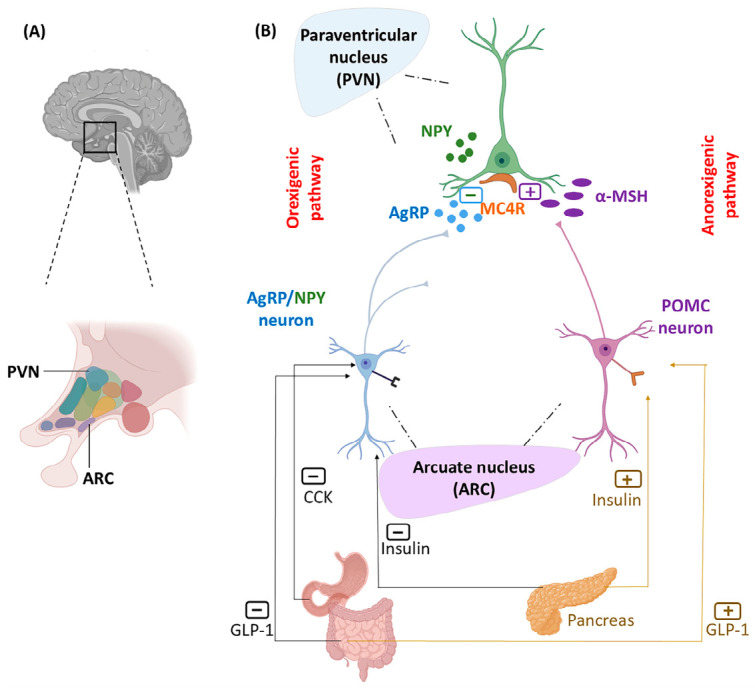
Orexigenic and anorexigenic pathways mediated by the melanocortin system [91]. (**A**) Hypothalamic paraventricular neurons (PVN) and arcuate nucleus (ARC). (**B**) Anorectic hormone α-MSH is secreted by POMC neurons that are located in the hypothalamic ARC in response to interaction with umami tastant. α-MSH interacts with MC4R located in PVN, which suppresses food intake. ARC houses other neurons, such as co-expressed ARgP and NPY, which trigger food intake by antagonizing the effect of α-MSH on MC4R. The peripheral hormones regulate appetite via the feedback mechanisms of CCK and GLP-1.

KEGG analysis identified pro-opiomelanocortin (*POMC*) as a major target of L10-352. POMC neurons in the hypothalamic arcuate nucleus (ARC) are central regulators of energy homeostasis. Tanycytes, as cells in the brain’s third ventricle, with access to CSF, express T1R1-T1R3 functional receptors. These cells are similar to type II cells in taste buds, and upon amino acid detection, release ATP. Secreted ATP from tanycytes activates POMC neurons [13]. In the ARC, anorexigenic neurons that synthesize POMC and cocaine-amphetamine related transcript (CART) counterbalance orexigenic neurons such as neuropeptide Y (NPY) and agouti-related peptide (AgRP) (Figure 6) [91]. POMC is cleaved into melanocortin peptides (ACTH, α-MSH, β-MSH, and γ-MSH), β-lipotropic hormone, and β-endorphin [92]. Melanocortin peptides modulate the secretion of cholecystokinin (CCK) and glucagon-like peptide-1 (GLP-1) via melanocortin receptors (MCRs) [91]. Therefore, POMC may mediate the satiety effects of umami peptides [93].

The T1R1-T1R3 receptor on intestinal I cells is activated by umami peptides, which leads to CCK secretion [12,94]. CCK mediates intestinal motility, gallbladder contraction, bile release, and pancreatic secretion [14]. NPY4R (pancreatic polypeptide receptor 1, PPYR1) responds preferentially to pancreatic polypeptide rather than NPY. NPY4R induction reduces appetite and modulates circadian food intake [95]. Pancreatic polypeptide, secreted postprandially, suppresses further food intake by activating satiety signals [96]. Therefore, NPY4R agonists are being explored for metabolic disorders. Gastrin-releasing peptide (GRP) is cleaved proteolytically to mature GRP and neuromedin. GRP regulates GI hormone secretion (e.g., gastrin) and transmits itch signals and scratching behavior [97]. Amino acid consumption and CCKR activation induce gastrin secretion. Gastrin release activates histamine secretion from enterochromaffin-like cells. Histamine then binds HRH2 (histamine receptor H2) on parietal cells to stimulate gastric acid secretion [98]. AGTII type 1 receptor (AGTR1) is co-expressed with T1R3 in mouse taste bud cells. Therefore, angiotensin II (AGTII) might have a role in umami taste sensation. AGTII can reduce food intake and is associated with weight loss by triggering GLP-1 and inhibiting NPY [99].

**Figure 7:**
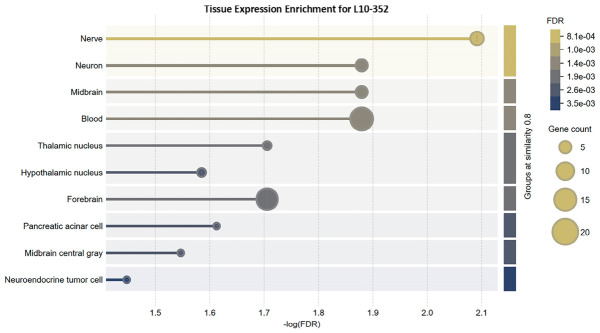
Tissue-specific gene expression pattern predicted for L10-352 umami peptide.

While satiety limits food intake, balanced feeding also depends on intact reward circuitry. Hypocretin receptors (HCRTR1/2) bind orexin A and B, neuropeptides expressed in the hypothalamus and periphery. The hypocretin neurons stimulate food consumption and reward processing. Loss or inhibition of HCRTR1 decreases palatable food intake in mice. Orexin-serotonin crosstalk balances ingestion and energy homeostasis [100]. Tissue expression pattern shows that 5-hydroxytryptamine (serotonin) receptor 2A (HTR2A) is located in the CNS (midbrain, forebrain, raphe nucleus) (Figure 7). The effect of serotonergic signaling in food intake depends on the regional expression of the receptor subtype [101]. Although increased central serotonin is generally associated with anorectic effects [102], *HTR2A* activation in the amygdala may promote feeding behavior [101]. Serotonin-HTR2A signaling stimulates opioid peptide release from the hypothalamic opioid neurons [103]. Besides the crosstalk between glutamatergic and serotonergic pathways, some HTR2A agonists improve memory and cognition [104,105].

Brain reward cascades are primarily regulated by dopaminergic and opioidergic pathways [103]. Dysregulation of these circuits has been linked to food addiction and eating disorders [106]. Glutamate neuron activation stimulates N-methyl D-aspartate receptor (NMDAR) on dopamine neurons, driving dopamine release and pleasure [107]. Dopamine receptor D4 (*DRD4*) and *DRD3* are targets modulated by L10-352. However, it has been reported that both hedonic response (liking) and incentive salience (wanting) to umami taste are primarily modulated by µ1 opioid receptor (OPRM1) network [108]. This aligns with *OPRM1* enrichment in GO analysis. The cannabinoid receptor 2 (*CNR2*) gene encodes the CB2 protein receptor expressed in brain areas associated with addictive-like behavior. Long-term consumption of palatable foods by mice resulted in CB2R-related food addiction. CBR2 inhibition has been proposed as a therapeutic target for uncontrolled eating disorders [109]. L10-352 is predicted to be a putative modulator of neuropeptide FF-amide peptide precursor (NPFF), which influences appetite, behavior, and endocrine function through dual anti- and pro-opioid actions [110].

Alpha-adrenergic receptors (ADRA1A and ADRA2B) are regulators of the sympathetic nervous system. While α1 receptors mediate vasoconstriction and smooth muscle contraction, α2 receptor activation decreases the sympathetic outflow. Agonists of α2 receptors aid behavioral control [111]. Noradrenaline reuptake inhibitors negatively affect taste sensation [112]. Noradrenaline is released in the taste bud synaptic cleft, and α1-adrenergic receptors are expressed on taste cells. However, evidence linking umami taste to adrenergic signaling remains limited [113]. Adrenergic activation in taste buds alters intracellular Ca^2+^ levels and may prolong depolarization [114]. α1 adrenoreceptor activation in the dorsal raphe nucleus reduces food ingestion and promotes satiety behavior in rats [115]. Adrenergic-glutamatergic crosstalk contributes to cognitive impairment in Alzheimer’s [116]. Reduced ADRA activity parallels NMDAR decline and requires further cell-specific studies [116].

Analysis of the biological process links L10-352 to chemokine signaling genes, including *CCL11, CXCL12, CXCR4, CCR3*, and *CCL5* (Table S1). CCL11 acts via CCR3 on neural cells [117]. While low CCL11 levels enhance neurogenesis and cognition, high levels reduce synaptic density and are linked to psychiatric disorders [118] and brain fog after COVID [119]. CXCL12 or stromal cell-derived factor 1 (SDF-1) interacts with CXCR4 and is involved in the innervation of taste buds and the GI, neural development, and axon guidance. CXCL12 regulates taste bud maintenance by promoting the differentiation of progenitor cells [120,121]. A low CXCL12 level has been observed in Alzheimer’s disease [122]. Despite its inflammatory role, CCL5 has been linked to hippocampal plasticity and spatial memory [123]. Collectively, L10-352 is potentially associated with interconnected neuropeptidergic pathways involved in feeding behavior and energy balance.

#### 3.2.3. L10-683 Umami Peptide Targets

**Figure 8:**
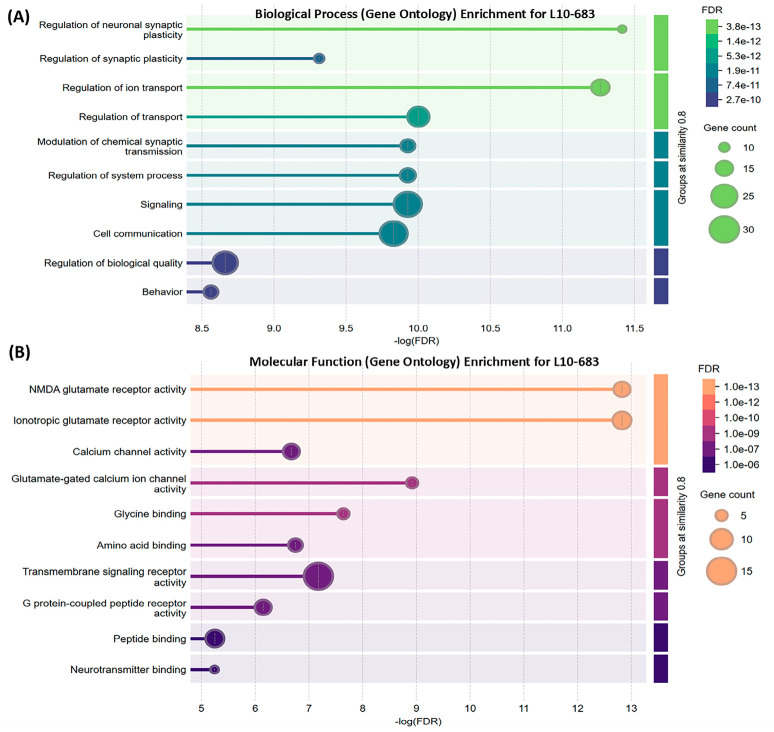
Functional enrichment visualization predicted for L10-683 umami peptide using the STRING V 12.0 database analysis mode. (**A**) Biological processes. (**B**) Molecular functions.

Neural synaptic plasticity, ion transport, behavior, and nervous system development are key biological processes associated with L10-683 targets (Table S1). Key targets include genes related to NMDA receptor and glutamatergic signaling (*GRIN3B, GRIN2D, GRIN2C, GRIN2A, GRIN3A, GRIN1, GRIN2B*), galanin (*GAL, GALR2*), incretin, metabolic, and appetite regulation (*GCG, GLP-1R, POMC*), as well as cholecystokinin (*CCK, CCKAR*), and somatostatin (*SST, SSTR*) pathways. NMDA receptors are composed of GRIN1 and GRIN2 (GRIN2A-D) subunits. *GRIN* genes regulate neural synaptic plasticity and glutamate receptor activity (Figure 8A,B). NMDA receptors are ionotropic glutamate receptors composed of obligatory GLUN1 subunits and regulatory GLUN2 subunits encoded by the *GRIN* genes [124]. NMDA receptors are essential for neural survival, memory formation, and long-term potentiation (synaptic strength). Glutamate binding opens NMDA-mediated Ca^2+^ channels that induce synaptic depolarization and activate transcription factors involved in neuron growth [125]. This process supports synaptic plasticity and new memory acquisition. Synaptic plasticity, which refers to the strengthening or weakening of synapses over time, is essential for learning and memory-related functions. However, prolonged depolarization results in excitotoxicity [126]. Although such adverse effects have not been reported for umami peptides, excessive glutamate exposure warrants caution [127]. L10-683 is predicted to target calcium/calmodulin-dependent protein kinase II alpha (CAMK2A) and Ras protein-specific guanine nucleotide-releasing factor 1 (RASGRF1). CAMK2A acts downstream of NMDAR to promote Ca^2+^ signaling, which is vital for the plasticity of glutamatergic synapses, long-term potentiation, and spatial learning [128]. RASGRF1 harbors a CAMK2A-binding domain and is involved in memory formation [129].

**Figure 9:**
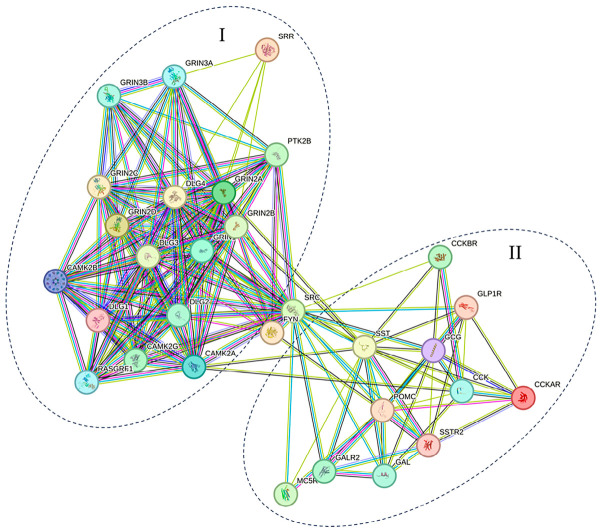
Predicted protein–protein interaction network using the STRING (version 12.0) database for L10-683 shows that glutamatergic targets are clustered together (**I**) and peripherally secreted hormones are also grouped separately (**II**) to form the gut–brain axis interactions.

Galanin (GAL) and galanin receptor type 2 (*GALR2*) are expressed in CNS regions linked to anxiety (Table S1). GALR2 signaling promotes neuronal plasticity and neurogenesis. However, the role of GAL in gastric motility remains uncertain [130]. KEGG analysis links L10-683 to gastric acid and insulin secretion (Table S1). Umami-rich foods slow gastric emptying and provide a fullness feeling [131], likely via GLP-1 release from intestinal L cells. The preproglucagon gene (*GCG*) is expressed in enteroendocrine L-cells, pancreatic α-cells, and NST neurons. *GCG* encodes glucagon, GLP-1, GLP-2, oxyntomodulin, and glicentin [132]. We observed co-expression of *GCG* and *GLP-1R* in functional enrichment analysis. Circulating GLP-1 binds GLP-1R on pancreatic cells to stimulate insulin secretion [133]. As a neuromodulator, GLP-1 stimulates intestinal nerve endings, triggering vagal nerve signaling to the CNS, and POMC-mediated appetite suppression [134]. γ-glutaminated beef protein hydrolysates enhance CCK and GLP-1 secretion and reduce TNF-α and IL-8 [135,136]. POMC-derived peptides act via melanocortin receptors; hence, MC5R agonists have been proposed to treat dry mouth disease [92]. L10-683 is linked to somatostatin (SST) secretion as well. SST-producing cells are present in the hypothalamus and mucosal D cells in the GI tract [137]. SST suppresses GI hormone release, gastric acid secretion, and motility. Of the five somatostatin receptor (SSTR) isoforms, SSTR2 and SSTR5 predominate in the endocrine system [138]. SSTR2 activation reduces gastric acid secretion, while SSTR5 stimulation inhibits GLP-1 production. Although SST plays dual orexigenic and anorexigenic roles, its effect on satiety is well-established due to slowing GI emptying and preventing hyperinsulinemia [91]. SRC and FYN lie at the intersection of two clusters in the L10-683 protein–protein interaction network controlling NMDAR function (Figure 9) [139].

#### 3.2.4. L10-1653 Umami Peptide Targets

GO analysis of L10-1653 indicates predominant targets are related to endothelin (EDN) signaling and axonogenesis regulation (*EDNRA, EDN3, EDN2, EDN1*), somatostatin pathway modulation (*SST, SSTR2*), renin-angiotensin system (*AGT*), cholecystokinin (*CCKAR, CCK*), and the POMC-melanocortin axis (*POMC, MC5R*). EDN peptide isoforms and receptors regulate vascular contraction and axon extension. EDNRA and EDNRB are the two main receptors of EDN. Excessive vasoconstriction capacity of EDN results in ischemia and nerve injury [140]. According to predictions, putative inhibition of EDN signaling by L10-1653 may be associated with pathways involved in axonogenesis. L10-1653 consumption is also linked to the *SSTR2* expression. SST downregulates *EDN1* [141], supporting the notion that a decrease in EDN1 level is concomitant with axon extension. KEGG analysis links L10-1653 with renin secretion and the angiotensinogen (AGT) pathway. Several studies report the ACEI effect of umami peptides from yeast extract hydrolysate, clam, or chicken soup [142,143,144]. ACE inhibitory activity of umami peptides has been associated with reduced EDN1 levels [144,145]. L10-1653 also acts via *POMC* and *MC5R* (Table 3). POMC also down-regulates *EDN1* [146]. Overall, L10-1653 may be linked to neurogenesis-related pathways through potential modulation of EDN signaling.

### 3.3. Targets of the Positive Control Umami Peptides

In addition to analyzing processes affected by novel umami peptides identified through artificial intelligence in this study, we have collected peptides whose umami taste has been validated by sensory panels and electronic tongues (Table 4). Functional enrichment analysis of these positive controls and the common targets with novel peptides is described within the following subsections.

#### 3.3.1. Umami Peptide from Fermented Soybean Product (Control 1)

Positive control 1 is a 10-mer umami peptide from tauco with an identical length to other bioactive peptides in this study (Table 4). The targets are mainly involved in neuropeptide signaling pathways, regulating hormone secretion, behavior, cognition, learning, and memory. The signaling processes span central (hypothalamic and cognitive) to peripheral (gut, immune, and metabolic) compartments. Some gene functions are involved in central regulation, such as cognition, reward, and appetite (*OXT, CHRM1, DRD4, GRM5, KISS1R, GHSR, TRHR*). Intermediate brain–gut peptide systems comprise *NTS, NTSR1, NTSR2, NMU, NMUR2*, and *NMUR1* encoding genes. Peripheral peptidergic regulation of GI motility and endocrine secretion is controlled by *MLN, MLNR, CCK, CCKAR, GRP, GRPR, GCG*, and *GCGR*. The inflammatory and immune interface targets are *TAC1, TACR2, TACR3, F2RL1*, and *KNG1*. Finally, the genes involved in lipid-derived mediator signaling, such as prostaglandins, leukotrienes, and lysophospholipids (*PTGER3, PTGFR, CYSLTR2, LPAR3, LPAR6, TBXA2R*), have been identified.

Taste can be a sensory stimulus for oxytocin secretion. Control 1 regulates *OXT*, which encodes a preproprotein called oxytocin-neurophysin I. Umami and oxytocin are processed in brain regions responsible for the reward process [147]. Oxytocin improves cognition, memory, learning, and food intake, consistent with the biological processes observed here [148,149]. KISS1 neurons in the hypothalamic ARC regulate metabolism through the crosstalk between kisspeptinergic and glutamatergic pathways [150]. Evidence supports the involvement of KISS1 neurons in food intake and energy expenditure [151]. While fasting reduces *KISS1* level, leptin or nutrient intake restores its expression [152]. *GHSR* encodes the growth hormone secretagogue receptor, also known as ghrelin (hunger hormone) receptor. While leptin suppresses appetite and ghrelin stimulates it, thyrotropin-releasing hormone (TRH) may trigger either orexigenic or anorexigenic signals. Arrival of umami inputs to the hypothalamic PVN, signaling protein-rich diet, activates TRH neurons and suppresses food intake [153].

The tauco-derived peptide targets the neurotensin (*NTS*) gene and its receptors, *NTSR1* (high affinity) and *NTSR2* (low affinity). NTS (cleaves into neurotensin and neuromedin N) is secreted by CNS and neuroendocrine cells [154]. Activation of the T1R1-T1R3 receptors in the GI tract induces NTS secretion, thereby mediating propulsive motility [155]. NTS-NTR1 activation in the small intestine also stimulates bile acid release. NTS and other NTR1 agonists influence cognition, spatial learning, and memory [156]. The role of NTS-expressing neurons in obesogenic behavior is pleiotropic and tissue-dependent, and warrants further study of their central and peripheral roles in appetite regulation [157,158]. NTS modulates various neurotransmitters in glutamatergic, dopaminergic, serotonergic, GABAergic, and cholinergic systems. These pathways regulate growth hormone synthesis, release, weight adjustment, and energy homeostasis [159]. The tauco-derived peptide affects neuromedin U (*NMU)* and its centrally located receptor *NMUR2*. This aligns NMU’s established role in feeding regulation. *NMUR2* knockout mice prefer an obesogenic diet, and NMU variants are involved in umami taste preferences in children [160,161]. MLN, a prepromotilin, is secreted from the small intestine and is cleaved into mature motilin. Motilin drives rhythmic contraction of intestinal smooth muscles and interdigestive GI motility [162]. The peptide hormone glucagon (GCG) and its receptor (GCGR) counteract the effect of insulin. GCG, secreted from pancreatic α-cells, promotes amino acid-driven gluconeogenesis and raises blood glucose [163].

Nociceptive fibers inside and around taste buds secrete the preprotein tachykinin 1 (TAC1) (Table 4) [164]. TAC secretion induces Ca^2+^ responses in taste cells, boosts umami perception, and agonists of TACR3 can reduce salt intake [165]. *TACRs* are also expressed on immune cells, such as B cells, and are involved in T-cell development and differentiation. TACs act as chemoattractants, promoting the migration of immune cells, NF-κB-mediated inflammation, IL-6, IL-8, TNF, COX-2, and PGE2 secretion, thereby affecting antimicrobial defense [166,167]. F2R-like trypsin receptor 1 (F2RL1) is a member of the protease-activated receptors [168]. *F2RL1* activation increases cytokines, such as IL-8, IL-1β, TNF-α, IL-4, IL-13, and IL-5, depending on tissue context [169].

#### 3.3.2. Umami Peptide from Chicken Soup (Control 2)

Control 2, the 9-mer umami peptide derived from chicken soup (Table 4), shares about 45% of targets with control 1, including *DRD4, OXT, GHSR, NTS, GRP, CHRND, NMU, CCKAR, TRH, NMUR1, TAC1, PTGER3, TACR2, CCK, GCGR, GCG, MLN*, and *TBXA2R*. Unique targets are promelanin concentrating hormone (*PMCH*) and its receptor (*MCHR1*). *PMCH* encodes a preprotein in the hypothalamus, which is cleaved into melanin concentrating hormone (MCH) and two other dipeptides. Elevated MCH levels or MCHR1 activity are linked to feeding behavior and energy balance [170]. *MCHR1* knockout mice display reduced ability in learning tasks and impaired long-term synaptic potentiation [171]. Conversely, activating MCH neurons during the REM phase of sleep disrupts hippocampus-dependent memory [172]. Arginine vasopressin (AVP), as a member of the vasopressin/oxytocin family, is synthesized in the hypothalamus, and its secretion decreases food intake (anorexigenic) [173]. AVP levels decrease after food intake and also suppress the NPY orexigenic pathway [174,175]. AVP neurons also regulate social recognition and memory [176,177]. Although hypothalamic AVP neurons receive gustatory input, most evidence comes from rodent studies [178]. These findings align with functional enrichment analysis of the chicken soup umami peptide, highlighting the most significant biological processes such as nervous system processes, eating behavior, memory, and learning (Table S2).

#### 3.3.3. Umami Peptide from Xuanwei Ham (Control 3 and Control 4)

**Figure 10:**
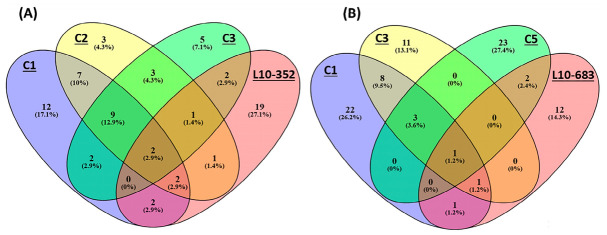
The intersection of predicted target genes involved in the most significant KEGG pathway (neuroactive ligand–receptor interaction) between novel identified umami ligands and control umami peptides drawn by the Venny 2.1.0 tool. (**A**) L10-352 has ten potential shared targets with the controls (C1, C2, C3). (**B**) L10-683 has five common targets with the controls (C1, C3, C5).

Ham is a favorable meat product for its distinct flavor (mainly due to the umami peptides). Control 3, an 8-mer umami peptide from Xuanwei ham (Table 4), targets neuropeptide and synaptic signaling pathways and feeding behavior (Table S2). Control 3 shares 13 and 15 common target genes (considering the most significant KEGG pathway) with control 1 and control 2, respectively (Figure 10A). Key shared target genes include *DRD4, OXT, KISS1R, CHRND, NMU, NMUR1, GRM5, PTGER3, PTGFR, LPAR6, CCK, GCGR, and GCG, HCRT, PMCH*, and *MCHR1*. Therefore, control 3 is associated with pathways related to food intake, appetite, satiety, and cognition primarily via the PMCH-MCHR1 axis, hypocretin (HCRT), CCK, and oxytocin signaling. Control 4, an 11-mer umami peptide (Table 4), influences neuroactive ligand–receptor interaction and insulin secretion (Table S2). Target genes in the KEGG pathway of control 4 include *OPRD1, NPFF, GLP1R, GCG*, and *OPRM1*. The opioidergic targets mediate hedonic response and incentive salience to umami taste [108]. Glucagon and GLP-1 production participate in the gut–brain axis to control feeding behavior after umami peptide intake [134].

#### 3.3.4. Umami Peptide from Jinhua Ham (Control 5)

The 6-mer umami peptide from dry-cured Jinhua ham (control 5) affects biological processes involved in the regulation of hormone secretion and behavior. The hub genes participate in the cephalic energy-balance module (*POMC, MC4R, MC3R, CALCR*), incretin-glucagon axis (*GCG, GLP1R, GCGR*), hypothalamic regulation (*VIP, SCTR, HTR7, CRHR2, AVP*), and adrenergic modulation (*ADRB1, ADRB2*). Major targets lie in the hypothalamic ARC, activating the anorexigenic pathway via POMC to MC4R in the PVN (Figure 6). Calcitonin receptors (CALCR) exert anorexigenic effects by modulating the melanocortin pathway. *CALCR* deletion in POMC neurons increases food intake [179]. *GCG* encodes proglucagon, which in pancreatic α-cells is processed into glucagon. In intestinal L-cells, GCG is processed by different prohormone convertases to GLP-1 [180,181]. GLP-1 activates GLP1-R on POMC neurons, contributing to reduced food intake. Corticotropin-releasing hormone (CRH) signaling via CRHR2 exerts a net anorexigenic effect by stimulating POMC and suppressing the NPY neurons in rodents. This mechanism has not been clearly demonstrated in humans. In contrast, human studies consistently link cortisol elevations to increased appetite, which highlights a divergence between central CRHR2 effects and peripheral HPA-axis responses [182]. Although arginine vasopressin (AVP) centrally modulates fluid retention, it has a significant role in feeding behavior as an anorexigenic factor [183]. AVP modulates insulin and glucagon release, linking to glucose metabolism. Vasoactive intestinal peptide (VIP) regulates gut motility and coordinates the central circadian feeding-fasting rhythm. VIP activates hypothalamic neurons, elevates *POMC* expression and α-MSH release, and thereby mediates anorexigenic effects [184,185]. Secretin (its receptor is SCTR) is another gut signal that is released in response to gastric acid and nutrients, suppressing hunger centrally. Growth hormone-releasing hormone (GHRH) primarily stimulates the release of growth hormone. GHRH induces food intake through both NPY/AgRP (orexigenic) and POMC (anorexigenic) pathways, depending on the nutritional state [186]. β1- and β2-adrenergic receptors (ADRB1 and ADRB2) mediate the effect of epinephrine and norepinephrine. Single-cell transcriptomics confirms the expression of adrenergic receptors in both POMC and AgRP neural populations. Electrophysiology in mouse ARC shows that noradrenalin excites NPY neurons via excitatory α1A- and β-adrenergic receptors and inhibits POMC through α2-adrenergic receptors [187]. KEGG analysis highlights salivary secretion pathways involving ADRAB1 and ADRB2 (Table S2), consistent with the positive correlation of β1 and β2 adrenergic receptors with cAMP-mediated salivation [188]. Among relaxin family receptors, only relaxin peptide family receptor 3 (RXFP3) and RXFP4 influence neuroendocrine control of feeding behavior. *RXFP3*, expressed in the brain and acts as an orexigenic regulator of hypothalamic neuroendocrine circuits. *RXFP4*-expressing neurons in the ventromedial hypothalamus modulate food intake and preference. RXFP1 and RXFP2 are peripheral, with no defined role in feeding [189,190]. Notably, the expression of various isoforms of adenylyl cyclase (*ADCY*) has been affected by control 5 (Table S2). ADCY converts ATP to cAMP, a general second messenger activating downstream transcription factors and ion channels. Certain ADCY isoforms are linked to immune function, metabolism, and memory [191]. In summary, Jinhua ham umami peptide signaling appears to be associated primarily with the POMC system, the central regulator of energy balance.

#### 3.3.5. Umami Peptide from Pufferfish Muscle Extract (Control 6)

Control 6, an 8-mer pufferfish umami peptide with low hydrophobicity (25%), affects neuroactive signaling and regulates hormone secretion, behavior, cognition, learning, and memory, similar to tauco peptide (control 1).

### 3.4. Intersection of Target Genes and Limitations of the Study

GO enrichment analysis identifies regulatory pathways that may not be reflected by mRNA changes alone. Key signaling events, such as protein–protein interactions or subcellular localization, occur without transcript variations [192]. Identifying overlapping biological processes or shared downstream effectors reveals integration nodes where distinct signaling cascades converge [193]. Different umami peptides may influence parallel pathways in distinct or the same compartments depending on the peptide’s structure, concentration, and route of administration. The neuroactive ligand–receptor interaction signaling is the most significantly affected KEGG category for both newly identified and validated umami peptides (Table 3, Table 4, Tables S1 and S2). We analyzed KEGG-derived target intersections among peptides to identify shared genes. L10-352 shares six targets with control 1 (*DRD4, GRP, EDNRB, GRPR, CCK, TBXA2R*), six common targets with control 2 (*DRD4, GRP, TBXA2R, HCRT, CCK, HCRTR1*), and five common targets with control 3 (*DRD4, NPFF, HCRT, HCRTR2, CCK*) (Figure 10A). L10-683 also shares targets with control 1 (*CCK, CCKR, GCG*), control 3 (*GCG* and *CCK*), and control 5 (*GLP1R, GCG*, and *POMC*) (Figure 10B). L10-1653 shares *CCK, CCKR*, and *POMC* target genes with other controls in the most significant KEGG pathway. Beyond the involvement of NPFF in opioidergic signaling and EDN in neurogenesis, these intersections suggest that the CCK/GLP-1/POMC anorexigenic pathway may represent a central regulatory axis associated with satiety following umami peptide intake. Overall, systems-level GO analysis reduces thousands of genes to a focused set of functional candidates. This efficiency reduces costs and accelerates validation via targeted assays [194].

Since the present findings are derived from computational prediction and functional enrichment analyses, the observed associations should be interpreted as hypothesis-generating. Although the systems-level approach enables identification of biologically coherent signaling networks, it does not provide direct evidence of gene expression changes or functional activation following umami peptide exposure. Future experimental validation is therefore required. Transcriptomic approaches such as RNA sequencing (RNA-seq) or microarray analysis could determine the up- or down-regulation of predicted target genes in relevant cellular or animal models following umami peptide stimulation. Such experiments would verify whether the computationally identified pathways are transcriptionally responsive to umami ligands. In addition, targeted validation using qPCR, Western blotting, ELISA, or receptor-specific functional assays would further clarify the mechanistic relevance of these predicted interactions. Thus, the current study provides a systems-biology framework that prioritizes high-value targets requiring future experimental investigation.

## 4. Conclusions

Umami peptides have been associated with reduced hunger, altered food preference, increased satiety, and cognitive-related outcomes in experimental settings. This study suggests the potential underlying molecular mechanisms of umami peptides using functional enrichment analyses. The most significantly modulated biological processes were related to feeding behavior, hormone secretion, neurogenesis (axonogenesis), and cytokine/chemokine signaling pathways. KEGG enrichment highlighted neuroactive ligand–receptor interaction. The results indicate an extensive crosstalk among glutamatergic, opioidergic, serotonergic, dopaminergic, cholinergic, adrenergic, and kisspeptinergic pathways. These interactions provide a framework linking peripheral and central pathways that may be involved in satiety and cognitive-related processes. These findings provide a rationale for further investigation of umami peptides as potential bioactive modulators. Current evidence suggests that physiological outcomes result from interconnected networks rather than a single linear pathway. Accordingly, the targets identified for umami peptides in this study must be interpreted within the broader regulatory axes in which they operate, including neural, endocrine, and immune signaling. Future studies should include experimental transcriptomic analyses or biochemical assays for each umami peptide.

## Figures and Tables

**Table 1:** Identification of novel umami peptides from marine bioactive sources using three accurate predictive models, namely UMPred-FRL (P1), Virtuous Multitaste% (P2), and Umami-MRNN (P3). Umami peptides were docked with the hT1R1-VFTD binding site. The number of interactions in the complex and the total interaction energy of molecular docking are reported. P: Predictor.

Peptide ID	Sequence	P1	P2	P3	Number of Interactions	Total Interaction Energy (Kcal/mol)
L10-40	DRKKFKNQSR	0.827	86	19.80	31	−137.1
L10-352	CKTEWIKSKC	0.98	87	10.74	20	−81.0
L10-683	KRKSNAERWR	0.702	82	7.96	28	−119.3
L10-1653	EKRKTCQRRW	0.992	87	34.41	25	−102.2

**Table 2:** Interactions between the umami peptide ligand (L10-40) and T1R1-hVFTD receptor are defined by the type of interaction, distance, and energy of interaction.

Ligand	Receptor Interacting Residue	Type of Interaction	Distance (Å)	Binding Energy (Kcal/mol)
N 1	Asp 218	H-donor	3.10	−7.8
N 10	Asp 218	H-donor	3.02	−2.3
CG 14	Asp 218	H-donor	3.35	−1.0
CG 15	Asp 218	H-donor	3.54	−1.5
NE 16	Asn 150	H-donor	2.94	−3.9
NH1 19	Asp 218	H-donor	2.79	−2.6
NH1 19	Asp 218	H-donor	2.81	−4.0
NH2 22	Asn 150	H-donor	2.73	−7.4
NZ 34	Gln 278	H-donor	2.58	−12.2
CG 69	Asp 147	H-donor	3.51	−0.5
NZ 72	Asp 147	H-donor	2.68	−7.3
NZ 72	Asp 147	H-donor	2.61	−7.8
CG 93	Glu 301	H-donor	3.58	−0.6
OG 105	Ala 170	H-donor	3.02	−1.4
NH1 118	Glu 301	H-donor	2.84	−4.7
NH1 118	Glu 301	H-donor	2.74	−5.8
NH2 121	Glu 301	H-donor	2.78	−4.7
C 124	Glu 70	H-donor	3.10	−2.9
O 88	Arg 277	H-acceptor	2.76	−5.4
O 125	Ser 385	H-acceptor	3.11	−0.5
O 125	Ser 385	H-acceptor	2.62	−0.8
O 125	Ser 384	H-acceptor	3.00	−0.5
N 1	Asp 218	Ionic	3.10	−3.8
NH1 19	Asp 218	Ionic	2.79	−6.0
NH1 19	Asp 218	Ionic	2.81	−5.9
NZ 72	Asp 147	Ionic	2.68	−7.0
NZ 72	Asp 147	Ionic	2.61	−7.7
NH1 118	Glu 301	Ionic	2.84	−5.7
NH1 118	Glu 301	Ionic	2.74	−6.4
NH2 121	Glu 301	Ionic	2.78	−6.2
NH2 121	Glu 301	Ionic	3.29	−2.8

**Table 3:** Biological process (BP) and KEGG enrichment results for the novel identified umami peptides using GO analysis. A false discovery rate (FDR) ≤ 0.001 is significant.

Peptide ID	GO Term Enrichment	FDR	Target Genes
L10-40	Defense response (BP)	1.87 × 10^−18^	*CCL17, CCL2, NFKB1, IRF1, IL1B, TP53, CCR2, CCR5, VCAM1, CCR1, FOS, CXCL8, ITGAL, JUN, CD40, SRC, HLA-A, ITGB2, RELA, IL6, CD44, TNF, IL10, CCR3, ITGAX, CCL5, CIITA, ITGAM*
L10-352	Neuroactive ligand–receptor interaction (KEGG)	1.74 × 10^−34^	*DRD4, GRP, NPFF, ADRA2A, SST, HCRT, SSTR2, AGTR2, NPY4R, CNR2, EDNRB, HRH2, EDN1, ADRA1D, GRPR, ADRA1A, DRD3, CCK, POMC, HCRTR1, ADRA2C, TBXA2R, OPRM1, HTR2A, LPAR2, LTB4R2, MC1R, HCRTR2, ADRA2B*
L10-683	Neuroactive ligand–receptor interaction (KEGG)	6.76 × 10^−20^	*GRIN3B, GRIN2D, GAL, SST, GRIN2C, CCKAR, GALR2, GRIN2A, SSTR2, GRIN3A, GRIN1, GLP1R, CCK, POMC, GCG, MC5R, GRIN2B*
L10-1653	Neuroactive ligand–receptor interaction (KEGG)	8.70 × 10^−16^	*SST, CCKAR, EDNRA, EDN3, SSTR2, AGT, EDN2, EDN1, CCK, POMC, MC5R*

**Table 4:** KEGG enrichment results of experimentally validated umami peptides by sensory panels or electronic tongues. A false discovery rate (FDR) ≤ 0.001 is significant. C: positive control umami peptide, UTT: umami taste threshold.

C	Sequence	Source	Validation	GO Term Enrichment	FDR	Target Genes
C1	DVFRAIPSEV	Tauco [27]	Sensory panel *	Neuroactive ligand–receptor interaction	5.68 × 10^−48^	*DRD4, OXT, MLNR, KISS1R, GHSR, NMUR2, NTS, GRP, CHRND, NMU, CYSLTR2, CCKAR, F2RL1, TACR3, TRH, NTSR2, NMUR1, GRM5, CHRM1, TAC1, PTGER3, NTSR1, LPAR3, PTGFR, TACR2, EDNRB, LPAR6, GRPR, CCK, GCGR, GCG, MLN, TBXA2R, AGTR1, TRHR, KNG1*
C2	AEEHVEAVN	Chicken soup [25]	Sensory panel (UTT: 0.33 mM)	Neuroactive ligand–receptor interaction	5.68 × 10^−48^	*DRD4, OXT, GHSR, MCHR1, NTS, GRP, CHRND, NMU, GRM1, HCRT, CCKAR, TRH, TACR1, NMUR1, GRM5, TAC1, PMCH, PTGER3, PTGFR, TACR2, AVP, CCK, HRH1, GCGR, HCRTR1, GCG, MLN, TBXA2R*
C3	RKYEEVAR	Xuanwei ham [28]	Sensory evaluation (UT: 0.40 mg/mL)	Neuroactive ligand–receptor interaction	1.93 × 10^−27^	*P2RY10, DRD4, OXT, KISS1R, MCHR1, CHRND, NMU, NPFF, NPFFR1, GRM1, HCRT, NMUR1, GRM5, NPFFR2, PMCH, PTGER3, KISS1, PTGFR, LPAR1, LPAR6, CCK, GCGR, GCG, HCRTR2*
C4	YVGDEAQSKRG	Xuanwei ham [28]	Sensory evaluation (UTT: 0.30 mg/mL)	Neuroactive ligand–receptor interaction	0.0000027	*OPRD1, NPFF, GLP1R, GCG, OPRM1*
C5	CCNKSV	Jinhua ham [29]	Sensory evaluation analysis and electronic tongue **	Neuroactive ligand–receptor interaction	6.03 × 10^−38^	*SCTR, DRD4, MC3R, PTGER2, VIPR2, RXFP2, MC4R, PTGER4, ADRB2, GHRHR, VIPR1, MC2R, HTR7, CRHR2, PTGER3, CALCR, VIP, ADRB1, GLP1R, GHRH, AVP, GCGR, POMC, FSHR, GCG, RXFP1, FSHB, PTH, MC1R*
C6	DPLRGGYY	Pufferfish [26]	Sensory panel (UTT: 0.27 mmol/L)	Neuroactive ligand–receptor interaction	1.81 × 10^−32^	*DRD4, OXT, MLNR, GHSR, NMUR2, NTS, GRP, CHRND, NMU, CYSLTR2, CCKAR, TRH, NTSR2, NMUR1, TAC1, PTGER3, NTSR1, LPAR3, PTGFR, LPAR6, CCK, GCGR, GCG, MLN, TRHR, KNG1*

* Taste dilution factor (DF) was reported for tauco fractions lower than 3 kDa and the umami intensity (umami-DF) was as high as 16–64. ** C5 produces a recognizable umami taste at 1 mg/mL (1.6 mM) but does not define a quantitative sensory threshold below this level.

## Data Availability

Data is contained within the article or Supplementary Materials.

## Supplementary Materials

The following supporting information can be downloaded at: https://www.mdpi.com/article/10.3390/nu18081299/s1, Figure S1: Molecular docking of the umami peptide ligands with T1R1-hVFTD and ligand–receptor interactions in the binding pocket (A) L10-352 (B) L10-683 (C) L10-1653; Table S1: Identification of target genes of novel identified umami peptides and gene ontology (GO) analysis results; Table S2: Identification of target genes of experimentally validated control (C1–C6) umami peptides and gene ontology (GO) analysis results.

## Abbreviations

The following abbreviations are used in this manuscript:

AGTR1	AGTII type 1 receptor
AGTII	Angiotensin II
ADRA	Alpha-adrenergic receptor
CAMs	Cell adhesion molecules
CCK	Cholecystokinin
END	Endothelin
F2RL1	F2R-like trypsin receptor 1
GAL	Galanin
GRP	Gastrin-releasing peptide
GLP-1	Glucagon-like peptide-1
GO	Gene ontology
GRAS	Generally Recognized as Safe
HCRTR	Hypocretin receptor
HTR2A	5-hydroxytryptamine receptor 2A
OPRM1	Mu opioid receptor
NMU	Neuromedin U
NTS	Neurotensin
NMDA	N-methyl-D-aspartate
PPYR1	Pancreatic polypeptide receptor
POMC	Pro-opiomelanocortin
SST	Somatostatin
TAC	Tachykinin
