# Supplementary figures and images for: The NeuroImmunoEndocrine Circuit of Umami Peptides: A Systems Biology Approach
Source: Nutrients. 2026 Apr 20;18(8):1299. doi: 10.3390/nu18081299 (PMC13118721; doi:10.3390/nu18081299)

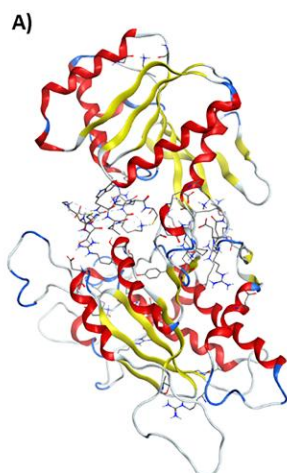

L10-352/T1R1-hVFTD

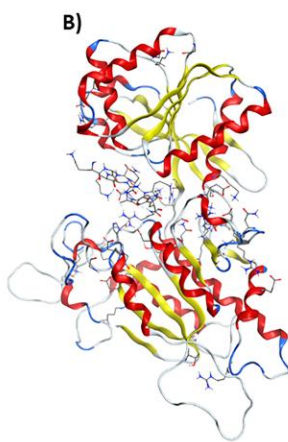

L10-683/T1R1-hVFTD

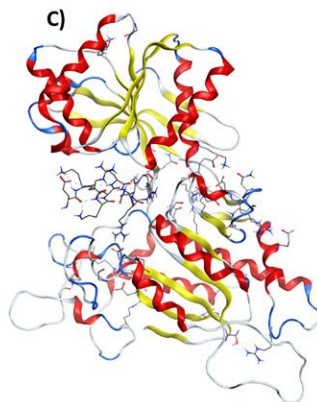

L10-1653/T1R1-hVFTD

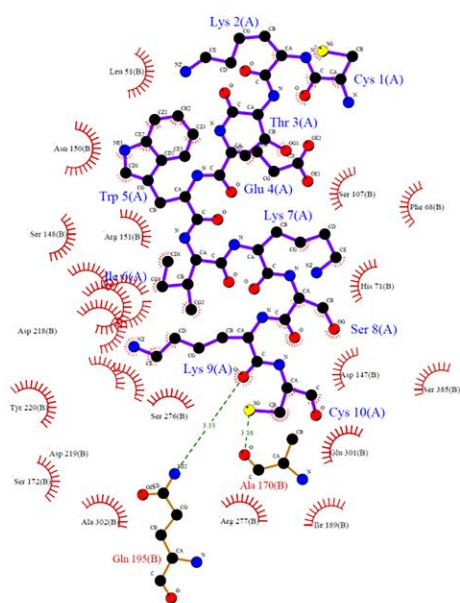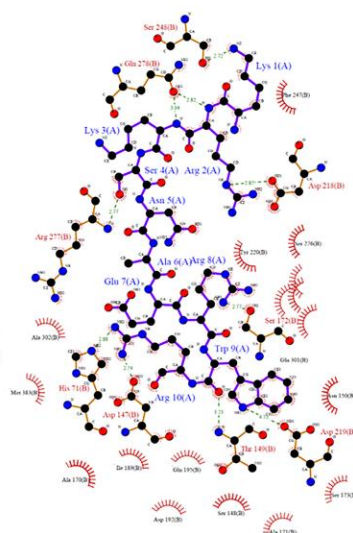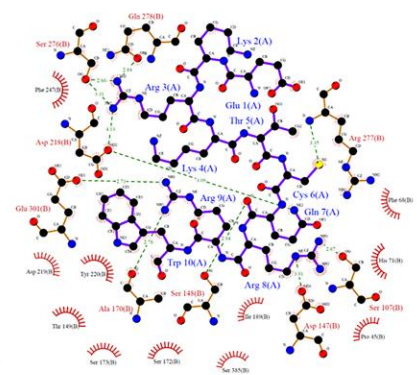

Supplement: Supplementary file 1 [file nutrients-18-01299-s001.zip › Figure S1.pdf]
